# PReS-FINAL-2158: Effect of canakinumab on functional ability and health-related quality of life in systemic juvenile idiopathic arthritis (SJIA) patients

**DOI:** 10.1186/1546-0096-11-S2-P170

**Published:** 2013-12-05

**Authors:** P Quartier, N Ruperto, N Wulffraat, H Brunner, R Brik, L Mccann, H Foster, M Frosch, V Gerloni, L Harel, C Len, K Houghton, R Joos, K Abrams, K Lheritier, S Kessabi, A Martini, D Lovell

**Affiliations:** 1 Necker-Enfant Malades Hospital, Paris, France; 2 PRINTO-Istituto Gaslini, Genova, Italy; 3 PRCSG, Cincinnati, OH, USA; 4 Novartis Pharmaceuticals Corporation, EH, NJ, USA; 5 Novartis Pharma AG, Basel, Switzerland

## Introduction

Canakinumab (CAN), a selective, fully human, anti-interleukin-1β monoclonal antibody, has been shown to be efficacious in SJIA patients (pts) in 2 phase 3 trials: Trial 1 (4-wk, randomized placebo [PBO]-controlled) and Trial 2 (open-label CAN treatment [Part 1] followed by a randomized PBO-controlled withdrawal phase [Part 2]). In Trial 1, statistically significantly more CAN than PBO group pts achieved an adapted pediatric ACR 30 response and in Trial 2, CAN allowed successful reduction/discontinuation of steroids and significantly reduced risk of flare. Safety profile of CAN was in line with expectations for a biologic agent in active SJIA. The persistent disabling features of SJIA and chronic pain can have a negative impact on health-related quality of life (hrqol). These outcomes were evaluated in the CAN phase 3 program.

## Objectives

To report pt-reported functional ability and hrqol outcomes from the CAN phase 3 program.

## Methods

The phase 3 analysis included 84 pts (CAN, 43; PBO, 41) in Trial 1 and 177 pts in Trial 2 (71 from Trial 1 entered) in Part 1, and 100 rolled into Part 2 (CAN, 50; PBO, 50). Pt-reported assessments included functional ability (as measured by the Childhood Health Assessment Questionnaire [CHAQ^©^]), pain (measured on a visual analog scale [VAS] of 0-100 mm as part of the CHAQ^©^), and physical (phs) and psychosocial (pss) health status in 5-18 year old pts., according to the Child Health Questionnaire-Parent Form (CHQ-PF50).

## Results

In Trial 1, CAN treatment was associated with a significant improvement in CHAQ disability score (estimated difference [ED] of -0.69 over time vs PBO in the least square mean [LSM] change from baseline [BL] [p = 0.0002]), which was ~3.6 × the minimal clinically important difference of -0.19. The LSM in overall pain intensity were significantly lower (both p < 0.0001) in the CAN group vs PBO both at Day 15 (ED, -46.42) and Day 29 (ED, -41.86). CHQ-PF50 phs and pss scores also showed significant improvements over time (ED CAN vs PBO in LSM change from BL, 12.07 and 7.28; p < 0.005 for both). Improvements in CHAQ disability, CHQ-PF50 phs and pss, and VAS pain scores were also observed in Trial 2 (Table 1).

**Table 1:** Patient- or Parent-reported functional ability and hrqol parameters in Trial 2

Outcome measure, mean (SD)	Baseline CAN, N = 177	End of Part 1 CAN, N = 177	End of Part 2 CAN, N = 50	End of Part 2 PBO, N = 50
**CHAQ disability score**	1.7 (0.8)	0.74 (0.9)	0.5 (0.9)	0.6 (0.8)
**Pain (VAS, 0-100 mm)**	66.6 (23.3)	20.2 (25.8)	13.6 (26.9)	17.0 (24.2)
**CHQ-PF50 phs score**	16.1 (14.3)	37.7 (17.2)	43.6 (17.4)	39.0 (18.1)
**CHQ-PF50 pss score**	41.6 (11.1)	50.7 (11.1)	53.6 (11.3)	52.7 (9.8)

Results are based on patients with both baseline and post-baseline values. N18 years old.

## Conclusion

Treatment with CAN demonstrated rapid, marked and continued improvement in patient-reported functional ability and hrqol of SJIA patients.

## Disclosure of interest

P. Quartier Grant/Research Support from: Abbvie, Chugai-Roche, Novartis, Pfizer, Consultant for: Abbvie, BMS, Chugai-Roche, Novartis, Pfizer, Servier, Sweedish Orphan Biovitrum, Speakers Bureau: Chugai-Roche, Novartis, Pfizer, N. Ruperto Grant/Research Support from: To Gaslini Hospital: Abbott, Astrazeneca, BMS, Centocor Research & Development, Eli Lilly and Company, "Francesco Angelini", Glaxo Smith & Kline, Italfarmaco, Novartis, Pfizer Inc., Roche, Sanofi Aventis, Schwarz Biosciences gmbh, Xoma, Wyeth Pharmaceuticals Inc., Speakers Bureau: Astrazeneca, Bristol Myers and Squibb, Janssen Biologics B.V., Roche, Wyeth/Pfizer, N. Wulffraat Consultant for: Novartis, Pfizer, Roche, H. Brunner Consultant for: Novartis, Genentech, Medimmune, EMD Serono, AMS, Pfizer, UCB, Jannsen, Speakers Bureau: Genentech, R. Brik Grant/Research Support from: Novartis, Consultant for: Novartis, L. Mccann: None declared., H. Foster Grant/Research Support from: Pfizer, biomarin, Speakers Bureau: Pfizer, M. Frosch: None declared., V. Gerloni: None declared., L. Harel: None declared., C. Len: None declared., K. Houghton: None declared., R. Joos: None declared., K. Abrams Shareholder of: Novartis, Employee of: Novartis, K. Lheritier Shareholder of: Novartis, Employee of: Novartis, S. Kessabi Employee of: Novartis, A. Martini Grant/Research Support from: Abbott, Astrazeneca, Bristol Myers and Squibb, Janssen Biologics B.V., Ely Lilly and Company, "Francesco Angelini", Glaxo Smith & Kline, Italfarmaco, Novartis, Pfizer Inc., Roche, Sanofi Aventis, Schwarz Bisciences, gmbh, Xoma, Wyeth Pharmaceuticals Inc.(Consulting fee). The GASLINI hospital which is a public hospital where I work as full time employee has received contributions to support the research activities of the network of PRINTO (http://www.printo.it), Consultant for: Abbott, Astrazeneca, Bristol Myers and Squibb, Janssen Biologics B.V., Ely Lilly and Company, Francesco A, Glaxo Smith & Klime, Italfarmaco, Novartis, Pfizer Inc., Roche, Sanofi Aventis, Schwarz Biosciences gmbh, Xoma, Wyeth Pharmaceuticals, Speakers Bureau: Astellas, Astrazeneca, Bristol Myers and Squibb, Glaxo Smith & Kline, Italfarmaco, medimmune, Novartis, D. Lovell Grant/Research Support from: NIH, Consultant for: Astra-Zeneca, Centocor, Jannsen, Wyeth, Amgen, Bristol-Meyers Squibb, Abbott, Pfizer, Regeneron, Hoffman La-Roche, Novartis, Genentech, Speakers Bureau: Genentech, Roche.

